# Biological therapy and surgery rates in inflammatory bowel diseases – Data analysis of almost 1000 patients from a Hungarian tertiary IBD center

**DOI:** 10.1371/journal.pone.0200824

**Published:** 2018-07-30

**Authors:** Kata Szántó, Tibor Nyári, Anita Bálint, Renáta Bor, Ágnes Milassin, Mariann Rutka, Anna Fábián, Zoltán Szepes, Ferenc Nagy, Tamás Molnár, Klaudia Farkas

**Affiliations:** 1 1^st^ Department of Medicine, University of Szeged, Szeged, Hungary; 2 Department of Medical Physics and Informatics, University of Szeged, Szeged, Hungary; University Hospital Llandough, UNITED KINGDOM

## Abstract

Inflammatory bowel diseases (IBD) [Crohn’s disease (CD) and ulcerative colitis (UC)], are chronic relapsing disorders of unknown etiology. The aim of this study was to determine demographic features, disease phenotypes, medical and surgical therapies in our IBD patients and to identify which parameters are in association with the need of surgery and/or biologic therapy. Data on demographic and clinical characteristics of the patients were analyzed from the IBD registry of the 1^st^ Department of Medicine, University of Szeged. The study period was between January 2007 and March 2015. Data of 911 IBD patients (428 CD, 483 UC) were analyzed. The median lag time between onset of symptoms and diagnosis proved to be significantly longer in UC than in CD (4.6 years vs. 2.1 years, p = 0.01). 40% of the patients received biological therapy, 301 patients underwent surgery required more frequently for CD than UC. Surgery was more common in CD patients with ileal location and penetrating behaviour. In UC, more severe disease onset predicted to unfavourable disease course. Higher proportion of surgery was shown in patient aged above 40 years in both CD and UC. Diagnostic delay of more than 1 year and appendectomy predicted to unfavourable disease outcome of both CD and UC. This analysis revealed that more than 1 year of diagnostic delay, disease activity at diagnosis in UC, CD, ileal location and penetrating behaviour are factors that may influence disease outcome. Use of thiopurines seemed to be protective in UC.

## Introduction

Inflammatory bowel diseases (IBD) [Crohn’s disease (CD) and ulcerative colitis (UC)], are chronic relapsing disorders of the gastrointestinal tract without known etiology. The diseases usually affect patients of younger age, and the risk of low quality of life due to chronic illness, together with hospital admissions and increased need of surgical interventions is associated with a higher risk of permanent work disability [1]. During the past decades, newer treatment paradigms incorporate more common and earlier use of immunosuppressive and biological therapies, which may lead to significant financial burdens of health care. The main role of these new therapeutic approaches is to favorably modify in the natural history of IBD, hopefully resulting in a decrease in complications and surgeries [2]. However, since the prevalence of IBD is expected to increase, direct and indirect costs to both health care system and society will also to be increased [3].

Descriptive epidemiologic studies and population-based cohort studies can provide valuable information about the burden of illness since identification of simple demographic and clinical factors associated with disabling disease has high importance in the management of IBD. However, only limited data are available from the Eastern Europe regarding to the rate of more complicated disease course, use of biological therapy and number of surgeries.

The **aim** of this study was to determine demographic features, disease phenotypes, medical and surgical therapies in our large IBD patients’ registry and to identify which parameters are in association with the need of surgery and/or biologic therapy as surrogate markers of worse disease course in CD and UC.

## Patients and methods

Patients diagnosed with IBD have been prospectively participated in an IBD registry launched at 1^st^ Department of Medicine, University of Szeged in 2007. This is large, prospectively maintained database that captures demographic and clinical data including intestinal and extraintestinal symptoms, disease activity assessed by validated activity scores, previous and current therapies, laboratory results and physiological parameters like blood pressure, heart rate, body weight and height, results of physical investigations, findings of endoscopies and imaging methods at every appointment of the patients. The registry includes both incident and prevalent cases. This database is suitable for the follow-up of patients since it records every visit of the patient with all of the important and necessary information detailed above.

In this study clinical data and descriptive statistics of the IBD outpatients were extracted and analyzed from the IBD registry of the 1^st^ Department of Medicine, University of Szeged between January 2007 and March 2015. Present age, age at diagnosis and at onset, smoking habits, history of appendectomy, the presence of familial IBD, the presence of extraintestinal manifestations, disease activities, number and type of surgeries, most important laboratory parameters and therapies were registered at every appointment of each patient. The diagnosis of CD and UC was based on the Lennard-Jones and later ECCO criteria [4]; disease phenotype was defined by the Montreal Classification [5]. Disease activity was assessed by the Crohn’s Disease Activity Index (CDAI) and the Mayo Scoring System in UC [6, 7]. On the basis of the activity scores, patients were divided in inactive, mildly, moderately and severely active groups. CRP was considered to be abnormal referring to active disease if being higher than 5 mg/l both in UC and CD. In the majority of the patients, first attendance at the 1^st^ Department of Medicine, University of Szeged was the same as the time of the diagnosis. Worse disease course was characterized by active disease with or without extraintestinal complications or perianal manifestations and the need of biological therapy and/or major surgery.

Differences in demographic and clinical characteristics, the presence of extraintestinal manifestations, number of surgeries and different types of treatments between CD and UC and predictors of outcomes in both diseases were analyzed statistically. Of therapies, corticosteroid, immunosuppressive and biological therapies were collected for statistical analysis.

### Statistical analysis

Statistical analysis was carried out using STATA 9.0 software. The Pearson's chi-square test, Wilcoxon ranksum test, Kruskal-Wallis test and logrank test were performed to analyze data. Median follow-up time with middle 50% interquartile range (IQR50%) were calculated. The symptom free follow-up periods were compared using Kaplan-Meier product limit estimator. A probability level of p<0.05 was considered to be significant.

### Ethical approval

This study was approved by the Clinical Research Coordination Center University of Szeged Faculty of Medicine Albert Szent-Györgyi Health Center. Number of ethical licence: 2640

## Results

### Demographic data and clinical characteristics of patient population with Crohn’s disease

428 CD patients diagnosed between January 2007 and April 2015 were included in the study. Male/female proportion was 194/234. Mean age at onset of symptoms and at diagnosis was 26.6±11.3 and 27.9±11.4 years. At diagnosis, ileocolonic and colonic locations and non-stricturing, non-penetrating behaviour were the most common (35.3%, 34.6% and 44.4%) disease phenotypes. Positive family history of IBD was reviewed in 8.8% of CD patients. Considering extraintestinal manifestations, arthralgia and arthritis occurred in 54% of the patients. The most common joints involved were knee, shoulder, hand, spine and hip.

### Demographic data and clinical characteristics of ulcerative colitis patient population

Data of 483 UC patients were analyzed in the study. Male/female proportion was 228/255. Mean age at onset of symptoms and at diagnosis was 30.3±12.4 and 30.9±12.5 years. Proctitis and left-sided colitis were the most frequent disease extents (34.2% and 34.6%). At the time of the first appointment, registered disease activity was mild in 41.6%, moderate in 41.6%, and severe in 12.2% of the UC patients. The remaining 4.6% of the patients was documented to have inactive disease at the time of first appointment. Positive family history of IBD was registered in 11.3% of UC patients. Arthralgia and arthritis were present in 57.8% of the patients.

### Proportions of biological therapy and/or surgery in CD

60.5% of the CD patients received thiopurine, 23.6% methylprednisolone, 45.6% biological therapy and 28% combined thiopurine and biological therapy. In CD, use of thiopurine was more common in patients with colonic and ileocolonic locations vs. ileal location (34.3% and 37.7% vs. 27.9%, p = 0.03). Need of biological therapy was not in association with any location or behaviour of CD and the presence of extraintestinal manifestations.

Surgery was needed in 228 patients (number of surgical interventions (both abdominal and perianal): 440). Bowel resection and perianal surgery was performed in 66% and 34% of the cases. Surgery proved to be more common in patients with ileal location compared to colonic and ileocolonic locations (p = 0.06) and with penetrating behaviour compared to non-stricturing, non-penetrating and stricturing behaviour (p≤0.001). Higher proportion (58.2%) of surgery was shown in patient aged above 40 years (p = 0.051). In CD, neither baseline CDAI or CRP were associated with the need of biological therapy and/or surgery.

### Proportions of biological therapy and/or surgery in UC

43.5% of UC patients were on thiopurine, 34.2% on methylprednisolone, 26.3% on biological therapy and 14.8% on combined thiopurine and biological therapy during the whole period. Use of thiopurines and biologicals did not show any association with disease extent.

Colectomy was performed in 77 patients throughout the follow-up period. 51% of the patients underwent laparoscopic surgery. One-, two,-and three-stage surgeries were performed in 13.3%, 40% and 46.7% of the operated patients. Significantly higher proportion (75.3%) of surgery was in patient aged above 40 years (p = 0.035). Disease extent did not show any association with the need of surgery. Rate of surgery did not differ significantly in patients receiving biologicals, however it was lower in patients treated with thiopurines (p = 0.045). Need of biological therapy and need of biological therapy and surgery together were more common in patients with more severe disease activity determined by pMayo score and CRP level at the first attendance (p≤0.001).

### Comparison of the demographic and clinical parameters of CD and UC patients

The median follow-up time of the patients was 3.6 years. Ratio of males/females did not differ significantly among the two disease groups (p = 0.435). The median lag time between onset of symptoms and diagnosis proved to be significantly longer in UC than in CD (4.6 years vs. 2.1 years, p = 0.01) (Table 1). However, after excluding cases where we were not able to perform a complete follow-up of the medical records, no significant difference remained regarding the median lag time between onset of symptoms and diagnosis.

**Table 1:** Median lag times between onset of symptoms and diagnosis in CD and UC.

Follow-up time (years)					
Group	No. of patients	Median	P25%	P75%	p-value
Total	911	3,6	0	9,6	<0,01
UC	483	4,6	0	10,3	
CD	428	2,1	0	8,6	
Non-zero follow-up time (years)					
Total	589	7,9	3,9	12,2	0,86
UC	349	8	3,7	12,7	
CD	240	7,7	4,2	11,9	

Smoking and history of appendectomy were significantly more common in CD vs. UC patients (p≤0.001 and p = 0.003). No difference was shown in the use of oral contraceptives between the two patient groups. No association was found between either positive family history of IBD, or any of the accompanying extraintestinal manifestations and any of the disease locations/extents or complicated disease behaviours in CD. Detailed demographic and clinical characteristics of the enrolled patients are summarized in Table 2 (S1 Table).

**Table 2:** Demographic and clinical characteristics of the enrolled patients.

	CD patients (n = 428)	UC patients (n = 483)
**Mean age at the present (years)**	41.2±13.8 (SD:0.67)	47.4±15.2 (SD:0.69)
**Mean age at diagnosis (years)**	27.9±11.4	30.9±12.5
**Mean age at onset of symptoms (years)**	26.6±11.3 (SD: 0.56)	30.3±12.4 (SD:0.58)
**Male/female ratio**	194/234	228/255
**Smoking habits (n, %)**		
- **Ex smoker**	109/385 (28.3)	148/448 (33)
- **Current smoker**	107/385 (27.8)	37/448 (8.2)
- **Never smoked**	169/385 (43.9)	263/448 (58.7)
**Appendectomy (n, %)**	76/378 (20.1)	26/448 (5.8)
**Use of oral contraceptives (n, %)**	119/372 (32)	146/437 (33.4)
**Location/extent (n, %)**		
- **Ileal**	110 (25.7)	NA
- **Colonic**	156 (34.6)	NA
- **Ileocolonic**	151 (35.3)	NA
- **Isolated upper GI**	11 (2.6)	NA
- **Proctitis**	NA	165 (34.2)
- **Left-sided colitis**	NA	167 (34.6)
- **Extensive colitis**	NA	151 (31.3)
** Behaviour (n, %)**		
** -Non-stricturing, non-penetrating**	190 (44.4)	NA
** -Stricturing**	101 (23.6)	NA
** -Penetrating**	136 (31.8)	NA
** -Perianal manifestation**	63 (14.7)	NA
** Extraintestinal manifestations (n, %)**		
** **- **Eye**	27 (6.3)	18 (3.7)
** **- **Skin**	55 (12.9)	69 (14.3)
** **- **Joint**	231 (54)	279 (57.8)

Ratio of males/females did not differ regarding the use of thiopurines and/or biological therapy. Rate of surgery proved to be significantly higher in CD vs. UC (p≤0.001). No relationship was found between smoking, appendectomy, positive family history of IBD and the presence of extraintestinal manifestations and the need of surgery or biological therapy. Use of oral contraceptives was protective on the need of surgery and biologicals in both diseases. Rates of surgery and biological therapy together were significantly more common in patients diagnosed more than 1 year after the onset of symptoms in CD and UC as well (p = 0.012 and p = 0.002).

## Discussion

The present study in which CD and UC patients were followed up for a median of 3.6 years has resulted in several important findings. Considering the whole study population, median lag time between onset of symptoms and diagnosis proved to be significantly longer in UC than in CD. 33.5% of the patients underwent surgery required more frequently for CD than UC. Biological therapy was given for 40%, immunomodulators for 53% of the patients. More patients needed surgery or biological therapy if they were diagnosed more than 1 year after the onset of symptoms. Surgery was more common in CD patients with ileal location and penetrating behaviour but did not show any association with disease extent of UC. Rate of surgery was lower in patients treated with thiopurines in UC. Higher proportion of surgery was shown in patient aged above 40 years in both CD and UC. Use of oral contraceptives was protective on surgery and the need of biological therapy and age above 40 years also proved to be protective on the need of biologicals in both diseases.

The overall prevalence and incidence of both CD and UC is increasing worldwide. Previous data suggested that approximately 2–14% of patients with CD and UC have a family history of IBD [8]. Our data revealed a bit higher proportion of IBD patients having positive family history of the disease. Smoking is one of the most consistently examined risk factors in IBD being associated with a more aggressive disease course in CD [9–11]. In our cohort, smoking did not prove to be a predictor of unfavourable disease outcome despite the proportion of current smokers was significantly higher in CD vs. UC. Appendectomy also shows different effects in CD and UC [12–14], however, our results could not confirm that appendectomy was associated with the need of biological therapy and/or surgery in CD and could not reveal protective effect in UC.

According to the results of the IBSEN cohort, 45% of the CD patients were diagnosed with colonic location and 60.5% with non-stricturing, non-penetrating behaviour at the time of diagnosis [15]. In UC, the initial extent of the disease was proctitis in 32%, proctosigmoiditis and left-sided colitis in 35% and extensive colitis in 33% [16]. In our study, the most common locations and behaviour of CD and the distributions of the different extents of UC were the nearly the same. An Australian study of Niewiadomski O et al. established a population-based registry to assess disease course in IBD [17]. Immunomodulators were prescribed in 57% of CD patients, and 19% with UC, steroids were used in 74% of CD and 63% of UC patients. Only 13% of CD patients were started on biological therapy. In our study, more than half of the patients received immunomodulators and more than one third of the patients received biological therapy during the whole follow-up period. Use of thiopurine was more common in CD patients with colonic and ileocolonic locations. Use of methylprednisolone seems to be lower in our cohort, although it also varies in the IBSEN studies-43% of the UC patients and 72% of CD patients had taken systemic glucocorticoids during the 5-year follow-up [15, 16]. In the Australian study, among CD patients, age < 25 years at diagnosis, ileocolonic and perianal disease were risk factors for biological therapy [17]. In our cohort, no relationship was found between location and behaviour of CD and disease extent of UC and the use of biological therapy, however need of biologicals was more common in patients below the age of 40 years and in UC patients with more severe initial disease activity.

In the IBSEN cohort, 28% of the CD patients underwent surgery with intestinal resection [15]. Intestinal resection rates were 26% at 5 years in the study of Niewiadomski et al. Risk factors of surgery included penetrating and stricturing disease and ileal involvement [17]. Colectomy rate in UC was 13% at 5 years with high CRP at diagnosis associated with colectomy [17]. According to the data of UC patients from IBSEN studies, overall colectomy rate proved to be 7.5% and 9.8% during the 5 and 10-year follow up periods [16, 18]. Initial presentation including extensive colitis, elevated ESR, anaemia and fever was significantly associated with an increased risk and age ≥50 years with a reduced risk of subsequent colectomy. In our cohort we found lower rate of surgery in patients treated with thiopurines. In CD, ileal location and penetrating behaviour, in UC, more severe initial disease activity (assessed by high CRP and pMayo score) and in both diseases age above 40 years at diagnosis related to surgery [16].

The systematic review and meta-analysis of Dias et al. highlights that delay in diagnosis is a crucial factor for unfavourable disease outcome in IBD, since due to delays in diagnosis, disease activity and bowel damage start to worsen in the lack of appropriate therapy [19]. A delay in confirming the diagnosis of IBD is associated with increased need for surgery, poorer treatment outcomes, reduced quality of life, and extent of disease [20, 21]. A French study revealed that diagnostic delay more than 12 months is associated with the presence of disease complications, suggesting that a delay in diagnosis beyond 12 months may result in missing the therapeutic window to intervene before disease complications occur [22]. Our results also suggest that more than 1 year diagnostic delay may relate to higher risk of surgery and need of biological therapy in CD and UC.

The major strength of the present study is the prospective inclusion and follow-up of incident IBD patients diagnosed in one of the largest IBD center of Hungary. The relatively high number of patients represents correctly the Hungarian IBD population. The patients are unselected and represent the whole spectrum of disease severity. Additionally, this study characterized a long follow-up period of both CU and CD patients However, as a limitation, there were cases in which we were not able to perform a complete follow-up of the medical records. After excluding these data from statistical analysis, no significant difference remained regarding the median lag time between onset of symptoms and diagnosis.

In this study we used objective measures, surgery and need of biological therapy as surrogate markers of worse disease course. Our follow-up study on almost 1000 IBD patients revealed that at diagnosis of IBD in a referral center, factors predictive of surgery were age below 40, although age above 40 years proved to be protective on the need of biologicals in both diseases as well as use of oral contraceptives. However, more than 1 year of diagnostic delay, disease activity at diagnosis in UC, CD, ileal location and penetrating behaviour seems also to be important factors that may influence disease outcome. Use of thiopurines seemed to be protective in UC.

Improvement of treatment strategies to minimize structural bowel damage and complications of disease has an increasing role in IBD. Hopefully the new trends in IBD management and the optimization of therapeutic options, combined with new drugs, will make possible to change the course of disease and provide better therapy and quality of life for patients suffering from IBD.

## Supporting information

S1 Table Demographic and clinical characteristics of the enrolled patients. There is no potentially identifying information in the supporting information file.
(XLSX)
